# Osteochondritis Dissecans Lesion of the Trochlear Groove: A Case of Nonsurgical Management for a Rare Lesion

**DOI:** 10.1155/2021/9776362

**Published:** 2021-12-13

**Authors:** Paul Krebs, Nicholas Walla, David Flanigan

**Affiliations:** ^1^Premier Health, Department of Family Medicine, Wright State University Boonshoft School of Medicine, 2400 Miami Valley Dr., Dayton, Ohio 45459, USA; ^2^Jameson Crane Sports Medicine Institute, The Ohio State University Wexner Medical Center, 2835 Fred Taylor Dr., Columbus, Ohio 43202, USA

## Abstract

Osteochondritis dissecans (OCD) lesions are potential causes of knee pain in pediatric patients, with lesions most frequently found on the lateral and medial femoral condyles. This case discusses an OCD lesion of the trochlear groove, a rare location for OCD lesions, in an 11-year-old female athlete. The patient presents after several years of knee pain that had acutely worsened, and both X-ray and MRI demonstrated the lesion, with MRI confirming a stable lesion. While previous literature has leaned towards surgical management, this patient was successfully managed nonoperatively in a locked knee brace for 12 weeks. She then went through 4 weeks of physical therapy and a 4 week progression back into soccer activity with return to full activity in 5 months.

## 1. Introduction

Osteochondritis dissecans (OCD) is a pathological process involving the osteochondral subunit that results in sequestration of the subchondral bone with or without disruption of the articular surface [1]. The etiology of this process has been hypothesized but is largely unknown. Pain and swelling are often the primary symptoms. Clicking, popping, and locking may also appear later in the disease course and can signal the presence of a loose body [2]. OCD lesions are often divided into juvenile OCD and adult OCD lesions [2]. The incidence of OCD lesions in children is higher than that in adults. While the overall age-adjusted annual incidence of OCD lesions is 6.09 per 100,000, numbers are higher in the pediatric population [3]. The joints involved most commonly include the knee, ankle, and elbow, with the incidence of lesions in these joints varying by age and sex.

Though more common in pediatrics, OCD lesions are seen in adults and can lead to pain and mechanical symptoms in the joints affected. In a study looking at patients 20–45, the incidence rate for OCD lesions was 3.42 per 100,000 person-years for all locations. The most common location for OCD lesions in this group was the ankle (2.08 per 100,000 person-years), followed by the knee (1.21 per 100,000 person-years) [4]. For adult patients, the risk of OCD lesions in men is twice the risk for women [4]. While the increased risk of OCD lesions for males is also present in children and adolescents, the locations of the lesions differ. In adolescents and children, the knee is the most common location, followed by the ankle and the elbow. Risk factors that may contribute to the development of OCD lesions include biological and mechanical factors such as endocrine disorders, genetic causes, vitamin D deficiency, obesity, joint anatomy, trauma, and overuse [5–7].

For those aged 6–19, the incidence of OCD lesions in the elbow is 2.2 per 100,000. The risk is even higher in the 12–19-year-old group (3.4 per 100,000) and in males compared to females (3.8 per 100,000 compared to 0.6 per 100,000, respectively) [8]. OCD lesions in the elbow are most commonly located at the capitellum (97.5 percent) followed by the trochlea and are also more common on the right side [8]. Sports typically associated with OCD lesions of the elbow include throwing sports such as baseball as well as sports requiring athletes to put significant force through the elbow such as gymnastics. The locations of lesion on the capitellum may differ between sports [9].

The incidence of OCD lesion in the ankle for patients aged 6–19 is 4.6 per 100,000 [10]. Unlike those in the elbow and the knee, the prevalence of OCD lesions for children in the ankle is higher in females than males, 6.0 per 100,000 compared to 3.2 per 100,000. Similar to that in the elbow, the rate was found to be higher in the 12–19-year-old age group, with an incidence of 6.8 per 100,000 patient-years [10]. The morphology and cause of ankle lesions can vary with the location in the ankle. Lateral lesions are most commonly seen following inversion injuries or trauma and are more shallow, while medial lesions are usually deeper and can be traumatic or atraumatic [11, 12]. Of these two locations, OCD lesions of the medial talus are the most common [13].

The most common location for OCD lesions overall in the pediatric population is the knee. The incidence of OCD lesions in the knee amongst patients 6 to 19 is 9.5 per 100,000, with males exhibiting a higher incidence than females (15.4 and 3.3 per 100,000, respectively) [14]. Additionally, the 12- to 19-year-old age group has a higher risk than the 6- to 11-year-old age group, 11.2 per 100,000 compared to 6.8 per 100,000, with males in the 12- to 19-year-old age group having the highest rate at 18.1 per 100,000 [14].

Given the prevalence of OCD lesions in children and adolescents, they are an important cause of knee pain for physicians to consider in pediatric patients. The appropriate diagnosis and treatment of a juvenile OCD lesion is crucial as there is a high risk of osteoarthritis if not appropriately managed [15, 16]. The age of the patient as well as the location, size, and stability of the lesion can impact treatment options and affect long-term prognosis. This case highlights an OCD lesion located on the trochlea of the femur, a rare location for these lesions [17, 18], treated nonoperatively in a 11-year-old female athlete.

## 2. Case Report

**Figure 1:**
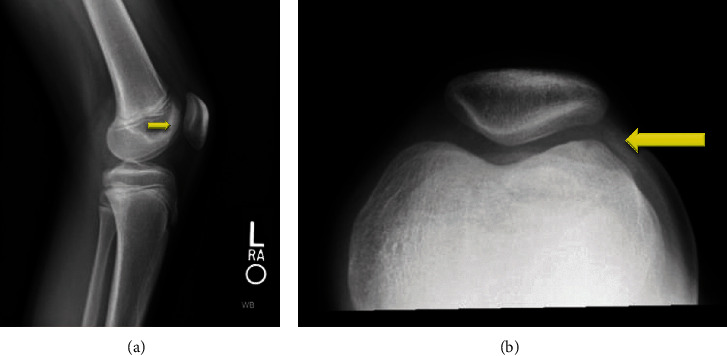
Initial X-rays of the patient showing osteochondritis dissecans (OCD) lesion of the lateral trochlear groove on (a) lateral and (b) sunrise views of the left knee.

**Figure 2:**
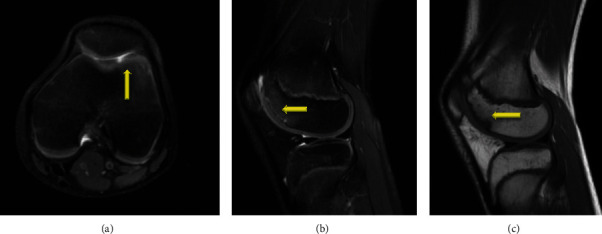
MRI evaluation of the knee showing stable trochlear osteochondritis dissecans (OCD) lesion on the (a) axial T2-weighted image, (b) sagittal T2-weighted image, and (c) sagittal T1-weighted image.

**Figure 3:**
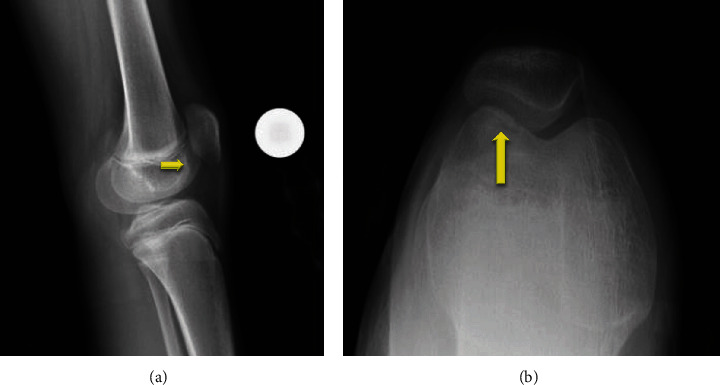
Follow up X-rays of patient with trochlear osteochondritis dissecans (OCD) showing bony healing on (a) lateral and (b) sunrise views of the left knee.

An 11-year-old female soccer player presented to the office with left knee pain that she first noticed 5 years ago after falling off a bike. Since that time she had a low level of pain in the knee, which was now significantly worse over the last month as soccer activities increased, the pain was worse with stairs, running, squatting, and kneeling; it was located in the anterior medial aspect of her knee. She denied radiation of the pain, numbness, tingling, popping, or locking. She had infrequent effusions and was using ice and NSAIDS as needed for pain. She had also tried a course of physical therapy with no improvement. An X-ray showed an osteochondritis dissecans (OCD) lesion of the lateral trochlear groove (Figure 1), and an MRI was obtained to stage the lesion. The MRI showed a stable OCD lesion of the trochlea (Figure 2). Given the stability of the lesion and patient age, the decision was made to proceed nonoperatively with weight bearing in a locked knee brace for 8 weeks for activities of daily living and restriction from athletics/sports. At her 8th week follow-up, she still had occasional pain with mild flexion and the X-ray showed bone formation of the trochlear groove without any subchondral collapse or loose bodies (Figure 3). She was continued in the locked knee brace for another 4 weeks. At her 12th week follow-up, she was pain free and was taken out of the knee brace. Physical therapy was started to strengthen her left lower extremity which had undergone atrophy in the brace, and by 16 weeks, she had regained her strength and was participating in soccer drills. She was transitioned from formal therapy to a home exercise program, and at 20 weeks, she was cleared to return to all activity, doing so without complication.

## 3. Discussion

OCD lesions can be seen in many joints and are a potential cause of knee pain in pediatric patients. The most common location for juvenile OCD lesions is in the lateral aspect of the medial femoral condyle [17]. The exact cause is unknown, but it is thought that microtrauma may play a role in the development of the lesion as patients are usually physically active individuals [19]. The typical pediatric patient presenting with an OCD lesion is a 12–19-year-old male with a reported incidence of 18.1 per 100,000 [14]. Symptoms often consist of vague or deep knee pain worse with activities, and unstable lesions can cause mechanical symptoms. Patients may report a history of trauma or injury, but OCD lesions can also be nontraumatic [20, 21]. Exam findings can be nonspecific and can include an effusion or tenderness over the involved cartilage and bone [19]. Wilson's test has been proposed as a diagnostic test but will not detect all OCD lesions [22]. Diagnostic imaging consists of X-rays and MRIs. Tunnel view X-rays are best for detecting lesions of the medial femoral condyle, but overall, X-rays may miss a large number of OCD lesions and are unable to stage lesions. Additionally, comparison views of the other knee are frequently needed to differentiate OCD lesions from ossification irregularities seen in pediatric patients [19]. MRI is more sensitive than X-ray and has the added benefit of providing information about stability which can then guide management [23, 24], though there is concern that in younger patients, MRI may not correlate as well with arthroscopic findings of stability [25].

Treatment of OCD lesions depends on the skeletal maturity of the individual and the joint involved, as well as the stability of the cartilage, location, and size of the lesion. Most skeletally immature patients with stable lesions of the knee can be treated conservatively with a combination of weight-bearing restrictions and activity modification, while unstable lesions will require surgery. Patients who fail conservative management may benefit from surgery [1, 19]. The location of the lesion also may impact treatment strategies. In a retrospective chart review of 192 patients, 28.7% of medial femoral condyle lesions progressed to needing surgery as did 40.3% of lateral femoral condyle lesions. For patellar lesions, 33.3% of lesions progressed to needing surgery. For tibial and trochlear OCD lesions in this pediatric population, 100% progressed to needing surgery; however, there were only 3 patients in this group with trochlear lesions [17]. Adolescents near skeletal maturity and adults will respond less frequently to nonoperative treatment and will frequently need surgery for OCD lesions, even for stable lesions [1, 19].

Similar to the knee, treatment of OCD lesions of the elbow depends on the fact that the physis is open or closed, the stability of the lesion, and the age of the patient. OCD lesions of the elbow in pediatric patients may have a slightly higher risk of progressing to surgery compared to the knee or ankle [26]. The cornerstone of nonsurgical treatment is rest from activity and has been shown to be very effective for stable lesions when the growth plate remains open and the patient has not started to lose motion. However, even with appropriate rest, some patients with stable lesions may progress on to needing surgery [27, 28]. Surgical management is typically recommended for unstable lesions, and older patients with closed physis are more likely to benefit from surgical intervention [27].

For the ankle, nonsurgical management usually consists of immobilization and nonweight bearing for 6 weeks, followed by gradual progression back to weight bearing and activities. Surgical interventions are similar to the knee and elbow and can include excision, debridement, bone marrow stimulation, osteochondral grafting, or autologous chondrocyte implantation [12]. Similar to the knee and elbow, outcomes of treatments for nonsurgical and surgical management at the ankle depend on the stage of the OCD lesion, its stability, the age of the patient, and the presence or absence of an open physis [12, 26].

The patient in this case highlights the potential success of nonoperative management in skeletally immature patients with OCD lesions. Moreover, she does so in an atypical presentation of an OCD lesion where there is limited prior literature on nonoperative management. This patient's OCD lesion was located in the trochlear groove, the rarest location for OCD lesions in the knee accounting for 0.6% of cases [14, 18, 29]. Prior retrospective studies have suggested that these lesions frequently progress to needing surgery [17]. Additionally, most juvenile OCD lesions are in 12–19-year-old males. Males have a 3.8 times greater risk of OCD lesions in the knees compared to females, and pediatric patients over 12 have a 3 times greater risk than children 6–11 years old [14]. While this single case does not suggest that all trochlear OCD lesions should be managed nonoperatively, it does highlight that this is a potential option for therapy in the appropriately selected patient. It also highlights the need for more knowledge in the literature regarding rare locations of OCD lesions such as the trochlear groove.

## Data Availability

The study is a case report and there is no underlying data.

## References

[1] Kocher M. S., Tucker R., Ganley T. J., Flynn J. M. (2006). Management of osteochondritis dissecans of the Knee. *The American Journal of Sports Medicine*.

[2] Friel N. A., Bajaj S., Cole B. J., Norman S. W. Articular cartilage injury and adult OCD: treatment options and decision making. *Insall & Scott Surgery of the Knee*.

[3] Pareek A., Sanders T. L., Wu I. T., Larson D. R., Saris D. B. F., Krych A. J. (2017). Incidence of symptomatic osteochondritis dissecans lesions of the knee: a population-based study in Olmsted County. *Osteoarthritis and Cartilage*.

[4] Weiss J. M., Shea K. G., Jacobs J. C. (2018). Incidence of osteochondritis dissecans in adults. *The American Journal of Sports Medicine*.

[5] Andriolo L., Crawford D. C., Reale D. (2020). Osteochondritis dissecans of the knee: etiology and pathogenetic Mechanisms. A Systematic Review. *Cartilage*.

[6] Bruns J., Werner M., Soyka M. (2016). Is vitamin D insufficiency or deficiency related to the development of osteochondritis dissecans. *Knee Surgery, Sports Traumatology, Arthroscopy.*.

[7] Kessler J., Jacobs J., Cannamela P., Shea K., Weiss J. (2018). Childhood obesity is associated with Osteochondritis dissecans of the knee, ankle, and elbow in children and adolescents. *Journal of Pediatric Orthopaedics*.

[8] Kessler J. I., Jacobs J. C., Cannamela P. C., Weiss J. M., Shea K. G. (2018). Demographics and epidemiology of osteochondritis dissecans of the elbow among children and adolescents. *Orthopaedic Journal of Sports Medicine*.

[9] Kajiyama S., Muroi S., Sugaya H. (2017). Osteochondritis dissecans of the humeral capitellum in young athletes. *Orthopaedic Journal of Sports Medicine*.

[10] Kessler J. I., Weiss J. M., Nikizad H. (2014). Osteochondritis dissecans of the ankle in children and adolescents. *The American Journal of Sports Medicine*.

[11] Canale S. T., Belding R. H. (1980). Osteochondral lesions of the talus. *The Journal of Bone & Joint Surgery*.

[12] Badekas T., Takvorian M., Souras N. (2013). Treatment principles for osteochondral lesions in foot and ankle. *International Orthopaedics*.

[13] Kramer D. E., Glotzbecker M. P., Shore B. (2015). Results of surgical management of osteochondritis dissecans of the ankle in the pediatric and adolescent population. *Journal of Pediatric Orthopaedics*.

[14] Kessler J. I., Nikizad H., Shea K. G., Jacobs J. C., Bebchuk J. D., Weiss J. M. (2014). The demographics and epidemiology of osteochondritis dissecans of the knee in children and adolescents. *The American Journal of Sports Medicine*.

[15] Sanders T. L., Pareek A., Johnson N. R. (2017). Nonoperative management of osteochondritis dissecans of the knee: progression to osteoarthritis and arthroplasty at mean 13-year follow-up. *Orthopaedic Journal of Sports Medicine*.

[16] Twyman R., Desai K., Aichroth P. (1991). Osteochondritis dissecans of the knee. A long-term study. *Journal of Bone and Joint Surgery. British Volume (London)*.

[17] Kessler J. I., Nikizad H., Shea K. G., Jacobs J. C., Ishkhanian R. M., Weiss J. (2013). The demographics, epidemiology, and incidence of progression to surgery of osteochondritis dissecans of the knee in children and Adolescents. *Orthopaedic Journal of Sports Medicine*.

[18] Smith J. B. (1990). Osteochondritis dissecans of the trochlea of the femur. *Arthroscopy*.

[19] Robertson W., Kelly B. T., Green D. W. (2003). Osteochondritis dissecans of the knee in children. *Current Opinion in Pediatrics*.

[20] Ewing J. W., Voto S. J. (1988). Arthroscopic surgical management of osteochondritis dissecans of the knee. *Arthroscopy*.

[21] Linden B. (1977). Osteochondritis dissecans of the femoral condyles. *The Journal of Bone and Joint Surgery. American Volume*.

[22] Wilson J. N. (1967). A diagnostic sign in osteochondritis dissecans of the knee. *The Journal of Bone and Joint Surgery. American Volume*.

[23] De Smet A. A., Fisher D. R., Graf B. K., Lange R. H. (1990). Osteochondritis dissecans of the knee: value of MR imaging in determining lesion stability and the presence of articular cartilage defects. *American Journal of Roentgenology*.

[24] Zbojniewicz A. M., Stringer K. F., Laor T., Wall E. J. (2015). Juvenile osteochondritis dissecans: correlation between histopathology and MRI. *American Journal of Roentgenology*.

[25] Uppstrom T. J., Gausden E. B., Green D. W. (2016). Classification and assessment of juvenile osteochondritis dissecans knee lesions. *Current Opinion in Pediatrics*.

[26] Weiss J. M., Nikizad H., Shea K. G. (2016). The incidence of surgery in osteochondritis dissecans in children and adolescents. *Orthopaedic Journal of Sports Medicine*.

[27] Takahara M., Mura N., Sasaki J., Harada M., Ogino T. (2007). Classification, treatment, and outcome of osteochondritis dissecans of the humeral capitellum. *The Journal of Bone & Joint Surgery*.

[28] Mourad F., Maselli F., Patuzzo A. (2018). Osteochondritis dissecans of the radial head in a young athlete: a case report. *The International Journal of Sports Physical Therapy*.

[29] Wall E. J., Heyworth B. E., Shea K. G. (2014). Trochlear groove osteochondritis dissecans of the knee patellofemoral joint. *Journal of Pediatric Orthopedics*.

